# Influence of implantations of extended depth-of-focus on standard automated perimetry

**DOI:** 10.1038/s41598-020-77214-8

**Published:** 2020-11-19

**Authors:** Jinhee Lee, Yosai Mori, Ryohei Nejima, Keiichiro Minami, Kazunori Miyata

**Affiliations:** Miyata Eye Hospital, 6-3, Kurahara-cho, Miyakonojo, Miyazaki 885-0051 Japan

**Keywords:** Medical research, Risk factors

## Abstract

This prospective study aimed to investigate the influence of an extended depth-of-focus intraocular lens (EDOF IOL) on standard automated perimetry. Ninety eyes of 90 patients who had undergone cataract surgery from February 2018 to December 2018 were included. No patients had any diseases that might affect the visual field. ZMB00 (+ 4.00 D add), ZXR00V (+ 1.75 D add), and ZCB00V (Johnson & Johnson Surgical Vision, Santa Ana, CA, USA) were used as multifocal, EDOF, and monofocal IOLs, respectively. Humphrey Visual Field 10–2 testing was performed 2–3 months after cataract surgery, acceptable reliability indices were measured, and mean deviation (MD), pattern standard deviation (PSD), foveal sensitivity and mean sensitivity (MS) were compared. Seventy-one eyes (ZXR00V: 24 eyes, ZMB00: 25 eyes, ZCB00V: 22 eyes) were used for the analyses. The MD and MS of the EDOF and monofocal groups were significantly higher than those of the multifocal group (*P* < 0.0051). However, the MD and MS of the EDOF and monofocal groups were not different (*P* > 0.23). The PSD and foveal sensitivity were not different among the groups. In non-glaucomatous patients, the MD and MS of the EDOF IOL were comparable to those of the monofocal IOL and better than those of the multifocal IOL.

## Introduction

Presbyopia correction intraocular lenses (IOLs), such as multifocal and extended depth-of-focus (EDOF) IOLs, have been implanted during cataract surgery to improve postoperative quality of vision and to reduce dependence on spectacles^1,2^. The in-focus light transmitted to the retina at the designed focus is reduced, and the out-of-focus light is superimposed onto the point spread function (PSF) ^3,4^. Consequently, the image contrast on the retina degrades, leading to lower contrast sensitivity and photic symptoms, such as glares and halos.

Recently, the influence on visual field sensitivity has also been addressed. The influence of diffractive multifocal IOLs^5–9^ was evaluated using several perimetries, such as frequent doubling technology^7^, Octopus^8^, Goldmann manual perimetry^9^, and Humphrey Field Analyzer (HFA; Carl Zeiss Meditec, Dublin, CA, USA)^5,6^. Some of these approaches demonstrated depressions of the hill of vision due to diffractive multifocal IOLs^5,6,9^, while the others did not show the depressions^7,8^. Recently, Aychoua et al. assessed the mean deviation (MD) and mean sensitivity (MS) of eyes after the implantation of diffractive multifocal and monofocal IOLs, and these parameters were measured in standard automated perimetry (SAP) using HFA with the Swedish Interactive Threshold Algorithm (SITA) standard threshold test algorithm under a 30–2 grid. The results revealed that eyes with multifocal IOLs exhibited lower values^6^. Similar results were obtained in the analysis using a 10–2 grid^5^. Although it is still unknown why the perimetry results are altered, a loss of light and a disturbed PSF inherent in diffractive multifocal IOLs are considered as the causative factors^3,4^. The previous findings suggested a risk of further reduction in contrast sensitivity. Thus, it is usually inadvisable to implant multifocal IOLs for glaucomatous eyes^5,10,11^.

A diffractive EDOF IOL with 1.75 D add power and an echelett grating has been developed and is commercially available^2^. The loss of light is 8%, which is lower than that of traditional diffractive multifocal IOLs (18%)^4^. Additionally, the PSF is less disrupted and closer to that of monofocal IOLs^3^. Hence, it was anticipated that the influence on perimetry results would be less than that observed with the use of diffractive multifocal IOLs. To the best of our knowledge, the influence of diffractive EDOF IOLs has not been assessed. The purposes of this study were to prospectively compare the perimetry results in eyes with diffractive EDOF IOLs with those in eyes with monofocal and multifocal IOLs.

## Results

Ninety eyes were enrolled. Due to insufficient reliability in the SAP measurements, 19 eyes were excluded from the analysis. Table 1 shows the demographic data of the three groups: group Mono with monofocal IOLs (ZCB00V, Johnson & Johnson Surgical Vision, Santa Ana, CA, USA), group EDOF with EDOF IOLs (ZXR00V, Johnson & Johnson Surgical Vision), and group Multi with multifocal IOLs (ZMB00, Johnson & Johnson Surgical Vision). The ages were significantly different among the three groups. No eyes showed relevant pathological characteristics in the SAP results.

**Table 1:** Demographic data in each IOL group.

	Multi group	EDOF group	Mono group	*P* value
N (eye)	25	24	22	
Male: female	6:19	11:13	5:17	
Right: left	16:9	19:5	14:8	
Age	69.7 ± 11.0	67.6 ± 5.4	72.0 ± 4.6	0.03**
	(33 to 83)	(58 to 77)	(64 to 79)	
Pupil size (mm)	4.8 ± 0.7	4.5 ± 0.7	4.9 ± 0.8	0.16*
	(3.6 to 6.2)	(3.1 to 5.8)	(3.5 to 6.5)	
Axial length (mm)	23.50 ± 0.99	23.59 ± 1.00	23.94 ± 0.86	0.28*
	(21.88 to 25.39)	(21.98 to 25.58)	(22.73 to 25.99)	
BCVA (logMAR)	− 0.14 ± 0.06	− 0.15 ± 0.07	− 0.12 ± 0.05	0.11**
	(− 0.18 to 0)	(− 0.18 to 0.10)	(− 0.18 to 0)	

Mean ± SD (range).

*EDOF* extended depth-of-focus, *Multi* multifocal, *Mono* monofocal, *BCVA* best-corrected visual acuity.

*ANOVA; **Kruskal–Wallis test.

**Figure 1:**
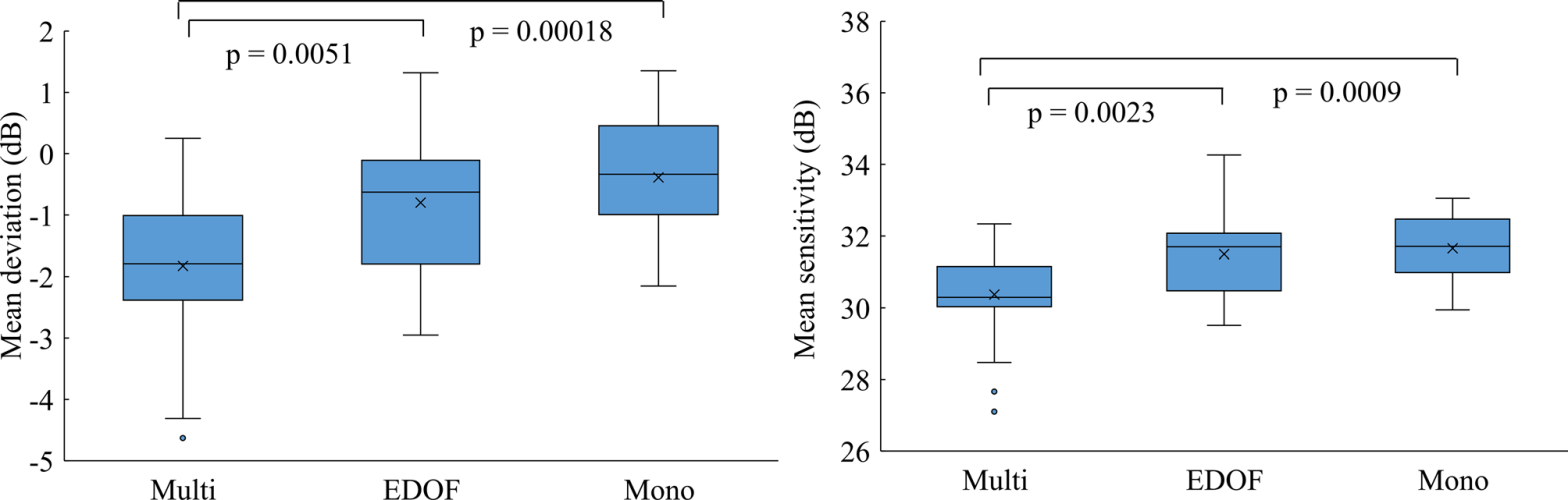
Comparisons of the mean deviation (MD) and mean sensitivity (MS) values on standard automated perimetry using the Swedish Interactive Threshold Algorithm standard threshold test algorithm under a 10–2 grid after the implantation of three types of intraocular lenses: multifocal (Multi group), extended depth-of-focus (EDOF group), and monofocal (Mono group). The MD and MS of the EDOF and Mono groups were significantly higher than those of the Multi group (*P* < 0.0051, t-test with Holm correction). However, the MD and MS of the EDOF and Mono groups were not different (*P* > 0.23, t-test with Holm correction).

**Figure 2:**
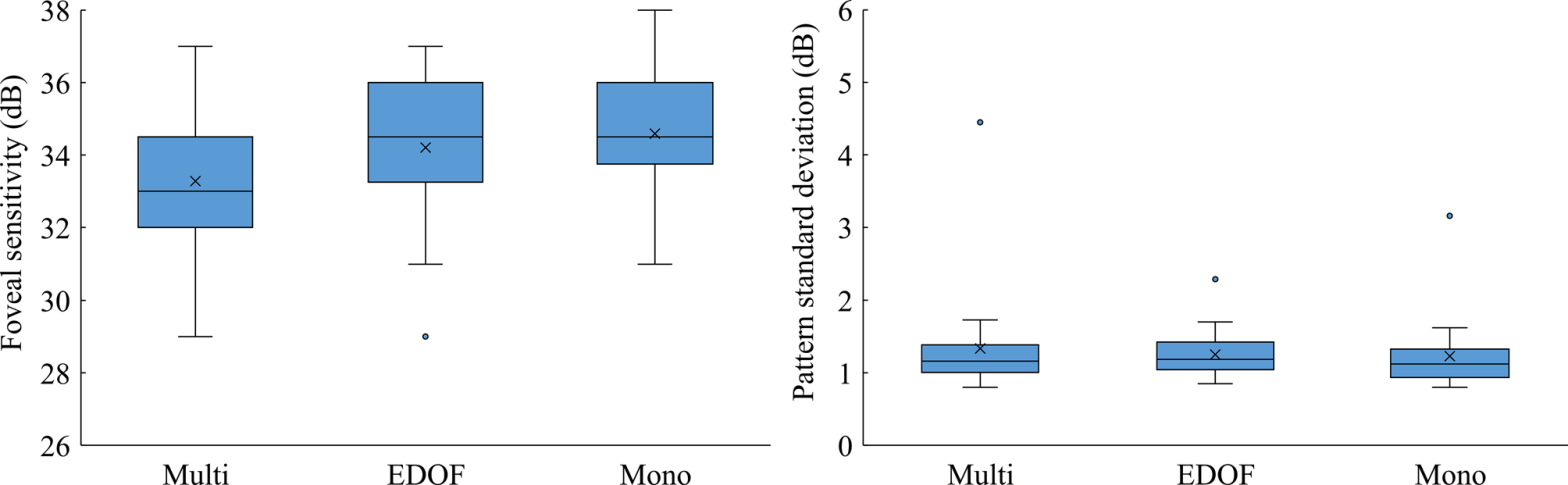
Comparisons of the pattern standard deviation (PSD) and foveal sensitivity values on standard automated perimetry using the Swedish Interactive Threshold Algorithm standard threshold test algorithm under a 10–2 grid after the implantation of three types of intraocular lenses: multifocal (Multi group), extended depth-of-focus (EDOF group), and monofocal (Mono group). The PSD and foveal sensitivity were not different among the three groups (*P* > 0.06, Wilcoxon signed-rank test with Holm correction).

Table 2 shows the mean values of the postoperative MD, pattern standard deviation (PSD), foveal sensitivity and MS after implantation of the IOLs, and Figs. 1 and 2 show the box plots. The Shapiro–Wilk test showed that the pupil size, axial length (AL), MD, and MS followed a normal distribution, while the age, best corrected visual acuity (BCVA), PSD, and foveal sensitivity did not follow a normal distribution. The mean MD and MS of the EDOF and Mono groups were significantly higher than those of the Multi group (*P* < 0.0051). However, there was no difference in the MD and MS between the EDOF and Mono groups (*P* > 0.23). The mean differences in the MD were 0.41 dB between the EDOF group and Mono group and 1.43 dB between the Multi group and Mono group. No difference was found in the mean PSD and foveal sensitivity among the groups (*P* > 0.06).

**Table 2:** Results of visual field testing in each IOL group.

	Multi group	EDOF group	Mono group	*P* value
MD (dB)	− 1.82 ± 1.24	− 0.80 ± 1.13	− 0.39 ± 0.96	< 0.001*
	(− 4.63 to 0.25)	(− 2.95 to 1.32)	(− 2.15 to 1.35)	
PSD (dB)	1.34 ± 0.67	1.25 ± 0.30	1.23 ± 0.48	0.45**
	(0.8 to 4.45)	(0.85 to 2.29)	(0.8 to 3.16)	
Foveal sensitivity (dB)	33.28 ± 1.87	34.21 ± 2.16	34.6 ± 1.78	0.045**
	(29 to 37)	(29 to 37)	(31 to 38)	
MS (dB)	30.37 ± 1.24	31.49 ± 1.16	31.66 ± 0.96	< 0.001*
	(27.10 to 32.33)	(29.51 to 34.26)	(29.94 to 33.05)	

Mean ± SD (range).

*EDOF* extended depth-of-focus, *Multi* multifocal, *Mono* monofocal, *MD* mean deviation, *PSD* pattern standard deviation, *MS* mean sensitivity.

*ANOVA; **Kruskal–Wallis test.

## Discussion

In the current comparison of SAP after the implantation of diffractive multifocal, diffractive EDOF, and monofocal IOLs, the Multi group had lower MD and MS than the other groups, and there was no difference between the EDOF and Mono groups. No difference was found in the PSD and foveal sensitivity. The differences in the MD between the Multi and Mono groups agreed with the previous evaluations using the HFA^5,6^, while the difference of 1.43 dB was less than previously observed results (1.87^5^ and 2.08^6^ dB). In the evaluations by Aychoua et al.^6^ the multifocal IOLs were mixed with silicone multifocal ZM900 (Johnson & Johnson Surgical Vision) and 809 M (AT LISA; Carl Zeiss Meditec), and SAP with a 30–2 grid was used. Farid et al.^5^ evaluated diffractive multifocal IOLs of ZMB00 and SN6AD1 (Alcon, Fort Worth, TX, USA), while the SAP settings were the same as those in the current study. In contrast, the current study compared the three types of IOLs that utilized identical platforms, except for presbyopia correction optics and tints. Hence, the significant changes in the MD and MS after the implantation of multifocal IOLs were confirmed.

The mean MD and MS in the EDOF group were higher than those in the Multi group, and there was no difference between the EDOF and Mono groups. The results suggested that the influence of EDOF IOL implantation would be comparable to conventional monofocal IOL implantation. Although it has not yet been determined why the MD and MS decrease after the implantation of diffractive multifocal IOLs, the optical degradation inherent to diffractive optics, such as increased loss of light and disrupted PSF, is considered a critical factor^3,4^. Optical-bench measurements showed that the loss of light and PSF of the EDOF IOL were similar to those of the monofocal IOL^3^. Clinically, Pedrotti et al. reported no difference in contrast sensitivity between monofocal and EDOF IOLs^12^. Although the EDOF and monofocal IOLs were tinted to block violet light, the tinting did not alter the SAP results^13^. Further investigations into the SAP results and the optical properties of EDOF IOLs are necessary.

There was no difference in foveal sensitivity. Sensitivity in the foveal area is crucial for patient vision. Flaxel et al. showed that there is a strong association between foveal sensitivity and best-corrected visual acuity (BCVA), and a foveal sensitivity of less than 30 dB would result in a BCVA less than 20/50^14^. The current results were above this threshold and the mean BCVAs of the three groups were beyond 20/20.

There were some limitations in this study. First, the visual field testing was performed only once. The results might fluctuate: the standard deviation of the MD values during five measurements was 0.54 dB in healthy subjects^15^. In addition, there was a learning effect^15^. Hence, we verified the examination using reliability measures, such as the fixation loss rate, false-positive rate, false-negative rate, and pupil size. Next, the influence of the SAP results on visual function was not evaluated. Although the decrease in the MD and MS was not significant, the influence on visual function was a concern. As shown in the clinical postoperative contrast sensitivity results, there was no difference^12^. However, there could be further slight changes in visual function, which may not be detected with the conventional contrast sensitivity test. Such slight changes could be evaluated using a contrast visual acuity chart or functional visual acuity test^16^. Last, the ages of the three groups were different. The influence on the MS of normal subjects was 0.58 dB per decade^17^, which might influence this result. However, the MD was a global index relative to normal subjects of the same age. Therefore, we believe that the difference in the MD would be insignificant in the current study.

In conclusion, the 10–2 grid SAP results demonstrated that the MD and MS after the implantation of diffractive EDOF IOLs were significantly better than those after the implantation of diffractive multifocal IOLs, and there was no difference with monofocal IOL implantations in normal subjects.

## Methods

### Subjects

This prospective comparative study was approved by the institute review board of Miyata Eye Hospital and was performed according to the tenets of the Declaration of Helsinki. Written informed consent was obtained from the patients before enrollment. This study was intended to test the hypothesis that the difference between the effects of EDOF and monofocal IOLs on the SAP results was less than the difference between the effects of multifocal and monofocal IOLs. Patients who had undergone cataract surgery with the implantation of diffractive multifocal IOLs, diffractive EDOF IOLs, or monofocal IOLs from February 2018 to December 2018 were recruited. The inclusion criteria were AL between 21 and 26 mm and intraocular pressure (IOP) lower than 21 mmHg. Eyes with any history of disease, ocular surgery, or trauma that might affect the visual field were excluded. In cases of bilateral surgery, the right eye was chosen for analysis.

The subjects were divided into three groups according to the implanted IOL: one-piece hydrophobic monofocal ZCB00V (Mono group), diffractive EDOF ZXR00V (EDOF group), and diffractive multifocal ZMB00 (Multi group). The number of eyes in each group was determined to be 13 or more. The minimum sample size was required to detect a difference in the MD values of 1.87 dB^5^ in the use of a noninferiority t-test and analysis of variance (ANOVA) with a significance level of 0.05 and detection power of 0.8 when the effect size was 0.958.

### Preoperative examinations

All the eyes underwent slit-lamp examination before surgery, and the normality of the anterior eye segment and fundus was confirmed. The AL and IOP were measured using an optical biometer OA-2000 (Tomey Corporation, Nagoya, Japan) and a noncontact tonometer FT-1000 (Tomey).

### Intraocular lenses

The implanted IOLs were one-piece hydrophobic and used the same platform: total length (13 mm), optic diameter (6 mm), 360-degree sharp edge, and anterior-shifted haptics. The multifocal ZMB00 was added 4.0 D for near vision, while the ZXR00V expanded the depth of focus using the echelett diffractive optics grating of a 1.75 D add power. The optic of ZMB00 was not tinted and that of ZCB00V and ZXR00V was tinted to block violet light.

Before surgery, the IOL type was chosen according to patients’ preferences for postoperative vision. For patients preferring far and near vision with no or reduced use of spectacles, the multifocal ZMB00 was recommended. ZXR00V was suggested when the patients required vision at intermediate distances and preferred to minimize the risk of photic symptoms. For other patients who were not interested in presbyopia correction or were uncomfortable with the photic symptoms associated with the use of ZMB00 and ZXR00V, monofocal ZCB00V was recommended. With sufficient explanations of the benefits and risks of the three types of IOLs, the choices of implanted IOLs were determined.

After topical anesthesia, the cataract was removed using a continuous curvilinear capsulorrhexis and phacoemulsification technique through a 2.2-mm superior sclerocorneal incision. The IOL was implanted in the capsular bag using the IOL injector system. The IOL power was calculated using the SRK-T formula, and the IOL power was targeted to be emmetropia. There were no complications in any of the surgeries.

### Postoperative examination

Two to three months after surgery, BCVA and standard automated perimetry were examined. The standard automated perimetry was measured using an HFA with the SITA standard threshold test algorithm under a 10–2 grid, white stimulus color, Goldmann size III target, and a background luminance of 31.5 apostilb. Refractive corrections for testing distance (33 cm) were performed for all the eyes. The reliability of the measurement was verified when a fixation loss rate lower than 15%, false-positive rate lower than 15%, false-negative rate lower than 20%, and pupil size larger than 2.5 mm were obtained.

The influence on the SAP was evaluated in the following indices: MD, PSD, foveal sensitivity, and MS^6^ within ten degrees of the center. The MS was calculated after taking the anti-log of the raw threshold values, averaging them, and converting the average to dB values^18^.

### Statistical analysis

Eyes with postoperative BCVA of 20/25 or worse were excluded from further analysis. Shapiro–Wilk tests were performed to confirm the normality of the demographic data and the perimetry results. For the parameters confirming a normal distribution, ANOVA following ad hoc pairwise comparison using a t-test with Holm correction was used for the intra-group comparisons. Otherwise, a Kruskal–Wallis test following ad hoc pairwise comparison using a Wilcoxon signed-rank test with Holm correction was used. Statistical analyses were performed using R version 3.5.1 (The R Foundation for Statistical Computing, Vienna, Austria). *P* < 0.05 was considered statistically significant.

## Data Availability

The datasets of this study are available from the corresponding author upon reasonable request.
